# (Con)text-specific effects of visual dysfunction on reading in posterior cortical atrophy

**DOI:** 10.1016/j.cortex.2014.03.010

**Published:** 2014-08

**Authors:** Keir X.X. Yong, Timothy J. Shakespeare, Dave Cash, Susie M.D. Henley, Jason D. Warren, Sebastian J. Crutch

**Affiliations:** Dementia Research Centre, Department of Neurodegeneration, UCL Institute of Neurology, University College London, London, UK

**Keywords:** Posterior cortical atrophy (PCA), Alzheimer's disease (AD), Acquired dyslexia, Crowding

## Abstract

Reading deficits are a common early feature of the degenerative syndrome posterior cortical atrophy (PCA) but are poorly understood even at the single word level. The current study evaluated the reading accuracy and speed of 26 PCA patients, 17 typical Alzheimer's disease (tAD) patients and 14 healthy controls on a corpus of 192 single words in which the following perceptual properties were manipulated systematically: inter-letter spacing, font size, length, font type, case and confusability. PCA reading was significantly less accurate and slower than tAD patients and controls, with performance significantly adversely affected by increased letter spacing, size, length and font (cursive < non-cursive), and characterised by visual errors (69% of all error responses). By contrast, tAD and control accuracy rates were at or near ceiling, letter spacing was the only perceptual factor to influence reading speed in the same direction as controls, and, in contrast to PCA patients, control reading was faster for larger font sizes. The inverse size effect in PCA (less accurate reading of large than small font size print) was associated with lower grey matter volume in the right superior parietal lobule. Reading accuracy was associated with impairments of early visual (especially crowding), visuoperceptual and visuospatial processes. However, these deficits were not causally related to a universal impairment of reading as some patients showed preserved reading for small, unspaced words despite grave visual deficits. Rather, the impact of specific types of visual dysfunction on reading was found to be (con)text specific, being particularly evident for large, spaced, lengthy words. These findings improve the characterisation of dyslexia in PCA, shed light on the causative and associative factors, and provide clear direction for the development of reading aids and strategies to maximise and sustain reading ability in the early stages of disease.

## 1 Introduction

Posterior cortical atrophy (PCA) is a clinico-radiological syndrome characterised by progressive visual impairment and parietal, occipital and occipito-temporal tissue loss. Most frequently a consequence of Alzheimer's pathology, PCA has been referred to as the visual variant of Alzheimer's disease, with a greater density of senile plaques and neurofibrillary tangles in the posterior cortices and fewer pathological changes in the prefrontal cortex and medial temporal areas relative to typical Alzheimer's disease (tAD) (Hof, Vogt, Bouras, & Morrison, 1997). The behavioural phenotype of PCA includes elements of Balint's syndrome (optic ataxia, oculomotor apraxia, simultanagnosia), Gerstmann's syndrome (agraphia, acalculia, left–right disorientation, finger agnosia) and limb apraxia with relatively spared episodic memory (Benson et al., 1988, Freedman et al., 1991, Levine et al., 1993, Ross et al., 1996).

Dyslexia is a common symptom of PCA (80–95%; McMonagle et al., 2006, Mendez et al., 2002) which presents early in the course of the disease, and patients frequently cite reading difficulties as being particularly debilitating. In everyday text reading (e.g., books, newspapers), patients often find spatial aspects of reading most challenging with frequent complaints of ‘getting lost on the page’. However, studies of reading in PCA have concentrated on single word reading and have described a number of patterns of dyslexia: neglect dyslexia (Catricala et al., 2011), attentional dyslexia (Saffran & Coslett, 1996), pure alexia (sometimes referred to as “letter-by-letter” – LBL reading) (Freedman et al., 1991, Price and Humphreys, 1995) and spatial alexia (Crutch & Warrington, 2007), with PCA patients also having difficulty reading cursive script (De Renzi, 1986) and nonwords (Mendez, 2001).

Most previous studies of dyslexia in PCA have been case studies. Consequently, group studies are required to gauge the extent and heterogeneity of reading dysfunction in PCA, and in particular to clarify the role of early aspects of visual function in influencing reading ability. The only group study of reading dysfunction in PCA to date employed flanked letter identification and single word reading tasks (Mendez, Shapira, & Clark, 2007). The flanked letter task revealed a significant effect of the visual similarity of flankers on target letter identification; unlike standard definitions of attentional dyslexia, this flanker effect occurred regardless of flanker category [numbers (e.g., 55S55), letters (e.g., KKXKK)]. The single word reading tests identified frequent visual errors in response to both regular and irregular words, an absence of regularization errors and disproportionate difficulty reading nonwords. These data led the researchers to suggest the term “apperceptive alexia” to reflect the contribution of deficits in visuoperception and visuospatial attention. The authors concluded that many aspects of reading dysfunction in PCA remained unexplained such as the potential contribution of a narrowing of the focus of spatial attention and suggested that analysis of reading speed and not just accuracy would be required to elucidate factors influencing reading performance.

The primary focus of the current study is upon the effect of perceptual variables on single word reading ability in PCA. Two perceptual attributes of words – inter-letter spacing and font size – merit particular consideration given previous evidence of their potential impact on reading in some individuals with PCA. First, the manipulation of inter-letter spacing in letter identification paradigms is well known to modulate the size of the so-called ‘crowding’ effect. Crowding is a perceptual effect in which the identification of target stimuli is inhibited by the presence of flanking stimuli irrespective of flanker category. Crowding is typically regarded either as a consequence of competition between a finite quantity of feature detectors (Townsend et al., 1971, Wolford and Chambers, 1984), or as resulting from excessive integration of features between flanker and target stimuli (Levi et al., 2002, Pelli et al., 2004). The crowding effect is diminished with greater spacing between target and flanker stimuli and exacerbated with increasing visual confusability between target and flanker. Crowding is implicated in reading dysfunction by previous observations that increased inter-letter spacing facilitates reading ability in dyslexics (Spinelli et al., 2002, Zorzi et al., 2012) and letter confusability predicts performance in LBL readers (Arguin et al., 2002, Fiset et al., 2005). In PCA specifically, spacing has been noted to improve performance in flanked letter identification tasks in several studies (Crutch and Warrington, 2007, Crutch and Warrington, 2009, Price and Humphreys, 1995). The most recent of these studies also showed an interaction between letter spacing and letter confusability in two PCA patients; at the word level, one of these patients demonstrated optimal reading with words with moderately spaced letters of lower summed confusability. If crowding is a component of dyslexia in PCA, this would raise the possibility that the conditions in which crowding effects are diminished in flanked letter identification tasks [increased spacing, reverse polarity flankers (Kooi, Toet, Tripathy, & Levi, 1994)] might be applied in order to facilitate whole-word reading.

The second perceptual attribute of particular interest in the current study is font size. Many PCA patients describe greater difficulty perceiving large than small objects (perhaps most strikingly by a patient who was unable to read the headlines of his newspaper but could read those of another passenger reading the same paper further down the train carriage on which he was travelling; see Crutch, 2013). Such ‘reverse size effects’ have been documented formally in a small number of patients with progressive visual disturbance who exhibited more impaired identification for large relative to small pictures, words and letters presented in isolation (Coslett et al., 1995, Saffran et al., 1990, Stark et al., 1997). This common clinical complaint in PCA has been attributed to a reduction in the effective visual field (Crutch et al., 2011, Russell et al., 2004). However the magnitude, prevalence and specificity of this effect in PCA remain unknown.

The presence of crowding and size effects in PCA patients who also exhibit poor reading is consistent with the predominant focus of atrophy in the parietal and occipital lobes which is associated with the syndrome (Lehmann et al., 2011, Whitwell et al., 2007). The neural correlates of crowding tend to be thought of as being in the occipital lobe, ranging from V1 to V4 (Anderson et al., 2012, Blake et al., 2006, Chung et al., 2007, Liu et al., 2009). A restricted effective visual field might result from damage to the superior parietal lobule or parieto-temporal regions, resulting in poor peripheral visual attention (Pierrot-Deseilligny et al., 1986, Russell et al., 2004), or damage to V6, resulting in disrupted peripheral field representations (Stenbacka and Vanni, 2007, Wandell et al., 2007).

The aim of the current study was to improve the characterisation of single word reading in PCA by manipulating the perceptual properties of words in a manner predicted to influence reading accuracy and speed. The perceptual properties examined included inter-letter spacing, font size, length, case, font type and confusability, and the performance of PCA patients was compared directly with that of tAD patients and healthy controls. It was hypothesised that perceptual properties would be a primary determinant of reading ability in the PCA but not tAD or healthy control groups. A secondary aim was to consider the role of early visual, visuoperceptual and visuospatial processing in PCA and tAD patients in order to improve our understanding of the causal and associative relationships between these different aspects of visual function and reading ability in PCA.

## 2 Methods

### 2.1 Participants

The study participants were 26 PCA patients, 17 typical AD patients and 14 healthy controls. The PCA patients all fulfilled clinical criteria for a diagnosis of PCA (McMonagle et al., 2006, Mendez et al., 2002, Tang-Wai et al., 2004) and research criteria for probable Alzheimer's disease (McKhann et al., 2011). The tAD patients fulfilled research criteria for a diagnosis of typical amnestic Alzheimer's disease (McKhann et al., 2011). All patient diagnoses were made based on clinical and neuroimaging data. The healthy controls were matched to the PCA and tAD groups on mean age and years of education, with the PCA and tAD participants additionally matched for mean disease duration and Mini-Mental State Examination score (MMSE; see Table 1). Ethical approval for the study was provided by the National Research Ethics Service London-Queen Square ethics committee and informed consent was obtained from all participants.

**Table 1:** Demographic information for the PCA, tAD and control groups. Means and standard deviations are presented for age, education, disease duration and MMSE.

	PCA	Typical Alzheimer's disease	Control
Number of participants	26	17	14
Gender (male/female)	10/16	12/5	5/9
Age (years)	61.4 ± 7.7	65.0 ± 5.1	62.7 ± 5.0
Education level (years)	14.6 ± 2.3	14.9 ± 2.4	16.1 ± 2.4
Disease duration (years)	4.4 ± 2.4	5.0 ± 1.7	–
MMSE^a^ (/30)	17.7 ± 5.0	17.5 ± 4.9	–

a Mini-Mental State Examination (MMSE: Folstein, Folstein & McHugh, 1975).

### 2.2 Reading assessment

#### 2.2.1 Perceptual corpus

All participants read aloud a total of 192 single words which involved simultaneous manipulations of five different perceptual properties:

• Inter-letter spacing (2 levels: no spaces and 2 blank s p a c e s).
• Font Size (2 levels: small and large): words were presented with a visual angle of letter height subtending .5° for small words versus 2° for large words.
• Case (2 levels: UPPER CASE and lower case).
• Length (3 levels: 3-, 5- and 7-letter words).
• Mean letter confusability (2 levels: high and low): upper case ratings for each letter were averaged from the confusability matrices of van der Heijden et al., 1984, Gilmore et al., 1979, Townsend, 1971, and Fisher, Monty, and Glucksbe (1969). Lower case ratings were averaged from the confusability matrices of Geyer (1977), and Boles and Clifford (1989).

The stimulus pool of 192 words was constructed from 24 8-word sets matched for mean frequency (CELEX: Baayen, Piepenbrock, & van Rijn, 1993), age of acquisition (AoA: Gilhooly & Logie, 1980) and concreteness (Coltheart, 1981) (see Table 3). The structure of the reading sets was such that the effect of each individual perceptual property upon reading performance could be directly compared as all other properties and variables were matched. For example, the font size effect could be readily examined as the small (*N* = 96) and large (*N* = 96) font words were matched for all background variables and contained an equal number of spaced and unspaced (*N* = 48 each), upper and lower case (*N* = 48 each), 3-, 5- and 7-letter words (*N* = 32 each) and high and low confusability words (*N* = 48 each).

**Table 2:** Neuropsychological scores of patients with PCA and tAD.

Test	Max score	Raw score			Norms/comment
		PCA (mean age: 61.0)	tAD (mean age: 65.0)	Difference	
**Background neuropsychology**					
Short Recognition Memory Test^b^ for words^a^ (joint auditory/visual presentation)	25	19.5 ± 3.7	14.7 ± 1.5	*p* < .0001	PCA: 5th–10th %ile, tAD: ∼<5th %ile (cut off: 19)
Short Recognition Memory Test for faces^a^	25	17.8 ± 4.0	16.8 ± 3.0	*p* > .3	Both ∼<5th %ile (cut off: 18)
Concrete Synonyms test^c^	25	20.0 ± 3.7	20.9 ± 2.5	*p* > .4	Both 10th–25th %ile
Naming (verbal description)	20	11.4 ± 6.6	13.7 ± 6.4	*p* > .2	Both ∼<5th %ile (cut off: 15)
Cognitive estimates^d^ (error score)	30	14.6 ± 7.5	10.6 ± 5.0	*p* = .074	Both ∼<1st %ile (cut off: 9)
Calculation (GDA^e^)^a^	24	1.6 ± 2.9	4.9 ± 5.3	*p* < .05	PCA: ∼<5th %ile, tAD: 5th–25th %ile
Spelling (GDST^f^ – Set B, first 20 items)^a^	20	8.9 ± 6.5	10.8 ± 5.6	*p* > .3	Both 10th–25th %ile
Gesture production test^g^	15	12.7 ± 3.4	14.1 ± 1.4	*p* > .1	–
Digit span (forwards)	12	6.0 ± 2.6	6.1 ± 1.4	*p* > .8	Both 25th–50th %ile
Max forwards	8	5.6 ± 1.8	5.5 ± .8	*p* > .9	–
Digit span (backwards)	12	2.6 ± 1.7	3.6 ± 1.9	*p* = .078	Both 5th–10th %ile
Max backwards	7	2.3 ± 1.3	3.3 ± 1.1	*p* < .05	–

*Psychomotor speed*					
A cancellation^h^: completion time	90 s	79.5 s ± 17.4	36.3 s ± 15.7	*p* < .0001	Both ∼<5th %ile (cut off: 32 s)
A cancellation^h^: number of letters missed	19	6.6 ± 5.1	.53 ± 1.1	*p* < .0005	–
CORVIST^i^ reading test	16	13.8 ± 3.0	15.7 ± .8	*p* < .05	–

**Visual assessment**					
*Early visual processing*					
Visual acuity (CORVIST): Snellen	6/9	(median 6/9)	(median 6/9)		
Figure-ground discrimination (VOSP^j^)	20	16.3 ± 3.0	18.6 ± 1.3	*p* < .01	PCA: ∼<5th %ile, tAD: 5th–10th %ile
Shape discrimination^k^	20	12.6 ± 3.9	17.2 ± 3.2	*p* < .0005	Healthy controls do not make any errors
Hue discrimination (CORVIST)	4	2.6 ± 1.1	3.0 ± 1.3	*p* > .3	–
Letters flanked by Numbers	24	20.1 ± 5.6	23.9 ± .2	*p* > .0005	Healthy controls do not make any errors
Letters flanked by Shapes	24	20.0 ± 4.5	23.9 ± .2	*p* > .0005	
Single letters (no flankers)	20	19.8 ± .61	20 ± 0	*p* > .2	

*Visuoperceptual processing*					
Object decision (VOSP)^a^	20	10.0 ± 4.1	15.9 ± 2.4	*p* < .0001	PCA: ∼<5th %ile, tAD: 10th–25th %ile
Fragmented letters (VOSP)	20	2.9 ± 3.9	13.5 ± 6.6	*p* < .0001	Both ∼<5th %ile (cut off: 16)
Unusual and usual views^l^: unusual	20	6.6 ± 6.8	9.9 ± 5.1	*p* > .1	Both ∼<1st %ile (cut off: 12)
Unusual and usual views^l^: usual	20	8.4 ± 5.5	16.5 ± 4.0	*p* < .0001	Both ∼<1st %ile (cut off: 18)

*Visuospatial processing*					
Number location (VOSP)^a^	10	1.8 ± 2.5	5.7 ± 3.8	*p* < .005	Both ∼<5th %ile (cut off: 6)
Dot counting (VOSP)	10	3.4 ± 3.2	8.1 ± 3.1	*p* < .0001	PCA ∼<5th %ile, tAD ∼5th %ile (cut off: 8)

a Behavioural screening tests supportive of PCA diagnosis.

b Warrington (1996).

c Warrington, McKenna and Orpwood (1998).

d Shallice and Evans (1978).

e Graded Difficulty Arithmetic test (GDA; Jackson & Warrington, 1986).

f Graded Difficulty Spelling Test (GDST; Baxter & Warrington, 1994).

g Crutch (unpublished).

h Willison and Warrington (1992).

i Cortical Visual Screening Test (CORVIST; James et al., 2001).

j Visual Object and Space Perception Battery (VOSP; Warrington & James, 1991).

k Efron (1969): oblong edge ratio 1:1.20.

l Warrington and James (1988).

**Table 3:** Different levels of reading variables for words from the perceptual corpus (*N* = 192) matched for AoA, concreteness and frequency.

Variable	Level	*N*	AoA	Concrete	Freq
Confusability	High	96	373	486	36
	Low	96	358	498	36

Spacing	Spaced	96	364	493	35
	Unspaced	96	367	491	37

Size	Large	96	365	491	37
	Small	96	366	493	35

Case	Upper	96	364	498	42
	Lower	96	367	486	30

Length	3	64	319	528	44
	5	64	357	499	32
	7	64	419	456	31

All words were presented in fixed random order, divided into two blocks with a break of approximately 20 min between blocks. All 192 words were presented in Arial Unicode MS.

#### 2.2.2 Cursive font reading

A subset (*N* = 12) of items were selected from the perceptual corpus fulfilling an equal number of levels of reading variables; these were re-presented in a cursive font (Wrexham Script) to 22 PCA patients, who were requested to read them aloud. The words were drawn from the no letter spacing condition and were presented in random order.

All words in the main and subsidiary reading experiments were presented for an unlimited duration at a viewing distance of 50 cm. Words were presented at the centre of the screen within a rectangular fixation box (22.5° in width, 4.3° in height); the fixation box remained on the screen throughout the experiment (including the inter-stimulus interval) to help maintain participant fixation within an area proximate to the word stimuli.

### 2.3 Background neuropsychology

PCA and tAD patients were administered a battery of background neuropsychological tests.

#### 2.3.1 Visual assessment

PCA and tAD participants completed a visual assessment examining three domains of visual processing:

##### 2.3.1.1 Early visual processing

a) Visual acuity test from the Cortical Visual Screening Test (CORVIST; James, Plant, & Warrington, 2001): task required discrimination of squares, circles and triangles at decreasing stimulus sizes corresponding to Snellen form acuity levels ranging from visual acuity of 6/9 to 6/36.
b) Shape detection test from the Visual Object and Space Perception battery (VOSP; Warrington & James, 1991): Figure-ground discrimination task involving random black pattern stimuli (*N* = 20), half with a degraded ‘X’ superimposed. Patients were requested to state whether an “X” was present.
c) Shape discrimination: The stimuli (*N* = 60) for this boundary detection task, adapted from Efron (1969), were a square (50 × 50 mm) or an oblong matched for total flux. There were three levels of difficulty: oblong edge ratio 1:1.63 (Level I), 1:1.37 (Level II), and 1:1.20 (Level III). The task was to discriminate whether each shape presented was a square or an oblong.
d) Hue discrimination (from the CORVIST): The stimuli (*N* = 4) comprised nine colour patches, eight of the same hue but varying luminance and one target colour patch of a different hue.
e) Crowding: Participants were asked to name letters under two conditions of spacing (condensed *vs* spaced) and flanked by numbers or shapes in two separate blocks of 24 trials.

##### 2.3.1.2 Visuoperceptual processing

f) Object Decision (from the VOSP): Stimuli (*N* = 20) each comprise four silhouette images, one of a real object (target) plus three non-object distractors.
g) Fragmented Letters (from the VOSP): Participants were asked to identify visually degraded letters (*N* = 20).
h) Unusual and usual views (Warrington & James, 1988): Participants were asked to identify photographs of real objects (*N* = 20) pictured from an ‘unusual’, non-canonical perspective. Items not identified from the non-canonical perspective are subsequently re-presented photographed from a more ‘usual’, canonical perspective.
i) Single letter naming: target stimuli were 20 alphabetic items (excluding I, J, O, Q, W and X) presented in isolation. Letters were presented in random order.

##### 2.3.1.3 Visuospatial processing

j) Number location (from the VOSP): Stimuli (*N* = 10) consist of two squares, the upper square filled with Arabic numerals in different positions, and the lower square with a single black dot. Participants were requested to identify the Arabic numeral whose spatial position corresponds to that of the target dot.
k) Dot counting (from the VOSP): Stimuli (*N* = 10) are arrays of 5–9 black dots on white background. Participants were asked to count the dots as quickly as possible without touching stimuli.

### 2.4 Data analysis

#### 2.4.1 Background neuropsychology

Differences between the PCA and tAD groups were calculated using a *t*-test.

#### 2.4.2 Behavioural covariates

Composite scores: All raw scores from the Visual Assessment were transformed into a standardised range (0–100) in which 0 and 100 corresponded to the minimum and maximum score achieved by any patient (irrespective of PCA and tAD group membership). Transformed scores in each visual assessment test were averaged within three visual processing domains in order to give composite scores for the following covariates of interest:

i) Early visual processing (Early): Shape discrimination, Figure-ground discrimination and Crowding (mean difference in accuracy for number and shape flankers between spacing conditions).
ii) Visuoperceptual processing: Object decision, Fragmented letters and Usual and Unusual views.
iii) Visuospatial processing: Number location and Dot counting

Composite scores were generated to include performance on different individual visual processing tasks in data analysis while restricting multicollinearity.

The raw scores for the following nuisance variables were also transformed into a standardised range for the PCA versus tAD regression analysis: Single letter accuracy, Digit Span (backwards), A Cancellation time (Willison & Warrington, 1992).

#### 2.4.3 Reading latencies

Reading latencies were manually determined from the onset of each word/letter using the digital audio editor Audacity (http://audacity.sourceforge.net). Latency data for erroneous responses and responses where participants had become overtly distracted from the task were removed from the analysis. Latency data greater than 2 standard deviations (SDs) from the mean of each participant were removed. Prior to latency regression analysis, latency data were transformed using a log transformation due to non-normal distribution of residuals.

In order to examine reading latency data we divided participants into 2 groups based on accuracy of reading words presented in a normal manner (small, unspaced words): As latency analysis was restricted to correct responses, reading latency data were difficult to interpret where there was a high error rate, resulting in a large proportion of missing data. For this reason, we divided participants into 2 groups based on accuracy of reading words under normal condition (small, unspaced words).

- Group 1 (PCA: *N* = 10, mean MMSE = 20.7, mean disease duration = 3.0 yrs; tAD: *N* = 16, mean MMSE = 17.7, mean disease duration = 5.1 yrs) made no errors on these items, or did not make enough reading errors to produce significant effects at the individual level using logistic regression or chi squared tests. The low proportion of errors allowed for analysis of latency data in this group.
- Group 2 (PCA: *N* = 16, mean MMSE = 16, mean disease duration = 5.8 yrs; tAD: *N* = 1, MMSE = 14, disease duration = 3.3 yrs) made enough errors to allow for meaningful error analysis. The high proportion of error prevented analysis of latency data in this group.

Accuracy data were analysed for both groups, meaning no participants were excluded from accuracy analysis; latency data analysis was restricted to group 1.

#### 2.4.4 Statistical analysis

Analyses of accuracy and latency data were conducted using logistic and linear mixed models respectively; both models used random subject effects and fixed effects of size, spacing, case, length, confusability, AoA, concreteness, frequency, orthographic neighbourhood size and word order, with the linear model of latency data also including accuracy rate as a fixed effect. Analysis of accuracy and latency data was carried out first on each of the PCA, tAD and control groups. Subsequently, group comparisons between PCA and tAD performance were conducted using similar logistic and linear mixed models but including only reading variables that were significant at the PCA and tAD group level, diagnosis and each of following behavioural covariates: Early visual processing, Early visual processing (excluding crowding), visuoperceptual processing, visuospatial processing, MMSE, Disease duration, digit span backwards, A cancellation, single letter naming. Differences in cursive font reading between PCA and tAD groups were calculated using a Wilcoxon rank-sum test and differences within groups were calculated using a Wilcoxon signed-rank test. The effects of interactions between neuropsychological performance and perceptual variables were analysed using logistic mixed models, including only reading variables which significantly predicted reading accuracy at the group level. Interaction analysis was restricted to accuracy data, owing to unequal numbers of responses for different levels of perceptual variables.

#### 2.4.5 Neuroimaging data

T1-weighted volumetric magnetic resonance (MR) images were acquired on a Siemens Trio TIM 3T scanner (Siemens Medical Systems) for 20 PCA patients. Images were acquired using a 3D magnetization prepared rapid gradient echo (MP-RAGE) sequence producing 208 contiguous 1.1 mm thick sagittal slices with 28-cm field of view and a 256 × 256 acquisition matrix, giving approximately isotropic 1.1 × 1.1 × 1.1 mm voxels; a 32-channel head coil was used.

For the voxel-based morphometry analysis, the MRI images were preprocessed using Matlab2012^®^ and SPM8 software (Statistical Parametric Mapping, Version 8; http://www.fil.ion.ucl.ac.uk/spm). Images were converted to NIFTI format (http://nifti.nimh.nih.gov) and rigidly re-aligned to standard space based on the international consortium for brain mapping template using the “New segment” function in SPM8. The standard space scans were segmented into grey matter, white matter and cerebrospinal fluid. The DARTEL toolbox (Ashburner, 2007) was used to perform inter-subject registration and normalising to MNI space, modulating the grey matter and white matter volumes according to the deformation fields and smoothing at 6 mm full-width half-maximum. Associations between regional grey matter volume and reading performance were assessed using voxel-wise linear regression models. Total intracranial volume, age, gender and MMSE score were included as covariates. Total intracranial volume was calculated by summing cerebrospinal fluid, grey and white matter volume. An explicit mask was applied to include voxels for which the intensity was >.1 in at least 80% of the images; this has been shown to reduce anatomical bias in participants with greater cortical atrophy (Ridgway et al., 2009). A voxel-wise statistical threshold of *p* < .05, family-wise error (FWE) corrected for multiple comparisons was applied in all analyses. In some figures, a more liberal threshold (*p* < .001 uncorrected) was applied for better visualization of additional areas where GM differences may be present.

## 3 Results

### 3.1 Reading assessment

#### 3.1.1 Perceptual corpus

##### 3.1.1.1 Overall summary

**Fig. 1:**
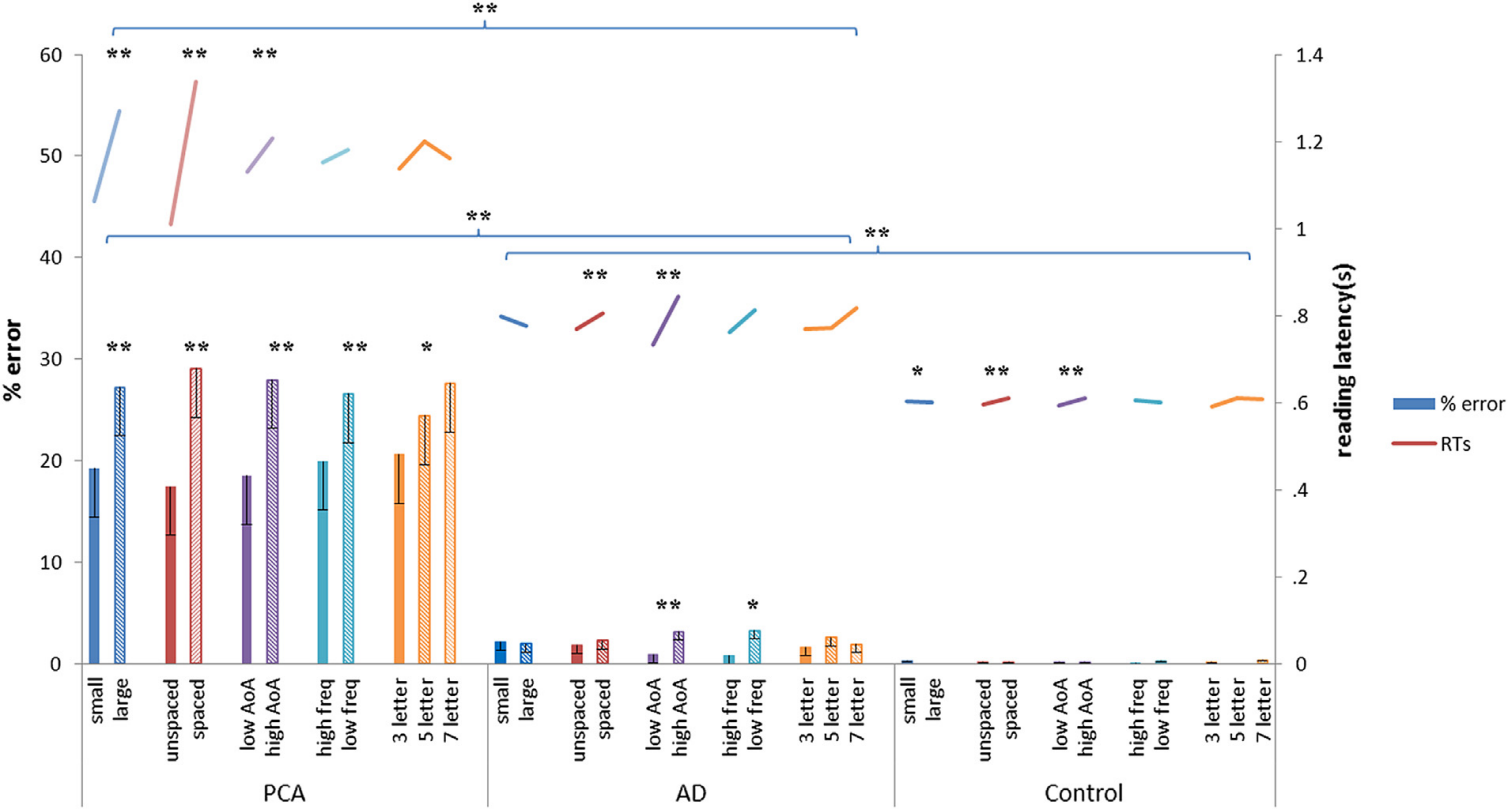
Summary of reading accuracy and latencies for the PCA, tAD and control groups. Asterisks denote a significant effect of each reading variable on reading speed or accuracy or significant differences between groups (*p < .05; **p < .005). Error bars show standard error for each group mean.

The mean percentage error rates and reading latencies are shown in Fig. 1. The PCA group was, on average, significantly less accurate and slower than both the AD group (*t* = 3.5, *p* < .005 and *t* = −2.8, *p* < .01, respectively) and the control group (*t* = 3.5, *p* < .005 and *t* = −3.2, *p* < .005, respectively). The AD group showed a trend towards being less accurate than the control group and was significantly slower (*t* = −2.0, *p* = .051 and *t* = 3.2, *p* < .005, respectively).

###### 3.1.1.1.1 Response accuracy in each group

PCA: PCA patients (*N* = 26; overall accuracy = 76.8%, SD = 47.1) were less accurate for words with increased inter-letter spacing (*z* = −10.2, *p* < .001), large font size (*z* = −7.9, *p* < .001), increased length (*z* = −2.8, *p* < .01), higher AoA (*z* = −6.9, *p* < .001) and lower frequency (*z* = 4.5, *p* < .001). There were also trends towards lower accuracy for words with greater orthographic neighbourhood size (*z* = −1.8, *p* = .077) and higher concreteness (*z* = −1.8, *p* = .084). There were no significant effects of case (*p* > .9), letter confusability (*p* > .3) or word order (*p* > .8) on accuracy. There were interactions between spacing and size (*z* = −2.17, *p* < .05) and spacing, size and length (*z* = −2.32, *p* < .05) with spaced, large words of increased length being read least accurately. There were interactions between spacing and frequency (*z* = −2.04, *p* < .05) and size and AoA (*z* = 3.73, *p* < .001) with spaced, low frequency words and large words of high AoA being read least accurately.

tAD: tAD patients (*N* = 17; overall accuracy = 98.0%, SD = 6.6) were less accurate for words with higher AoA (*z* = −4.5, *p* < .001), lower frequency (*z* = 2.6, *p* < .01) and for words which were read later in the assessment (*z* = −2.8, *p* < .01).

Controls: There was no effect of any of the variables on reading accuracy at either the group level (*N* = 13; overall accuracy = 99.8%, SD = .04) or individual level.

###### 3.1.1.1.2 Reading latency in each group

PCA: PCA patients (*N* = 10; overall mean reaction time (RT) = 1.17 sec, SD = .56) were slower to read words with increased inter-letter spacing (*z* = 11.8, *p* < .001), large font size (*z* = 5.8, *p* < .001), and higher AoA (*z* = 4.4, *p* < .001). Overall reading accuracy was also a significant predictor of reading speed (*z* = −3.9, *p* < .001).

tAD: tAD patients (*N* = 16; overall RT = .73 sec, SD = .16) were slower to read words with increased inter-letter spacing (*z* = 4.8, *p* < .001) and higher AoA (*z* = 4.4, *p* < .001) that were read earlier in the assessment (*z* = −2.9, *p* < .005). There was a trend towards words of lower frequency being read more slowly (*z* = −1.8, *p* = .073). Overall reading accuracy was also a significant predictor of reading speed (*z* = −3.9, *p* < .001).

Controls: The control group (*N* = 14; overall mean RT = .59 sec, SD = .08) were slower to read words with higher AoA (*z* = 5.1, *p* < .001), increased inter-letter spacing (*z* = 3.3, *p* < .005), lower letter confusability (*z* = −2.6, *p* < .01), decreased font size (*z* = −2.0, *p* < .05) that were read earlier in the assessment (*z* = −8.2, *p* < .001). There was also a trend towards smaller words being read more slowly than larger words (*z* = −1.9, *p* = .055). Overall reading accuracy was also a significant predictor of reading speed (*z* = −2.4, *p* < .05).

##### 3.1.1.2 Between-group comparisons (PCA *vs* tAD)

**Fig. 2:**
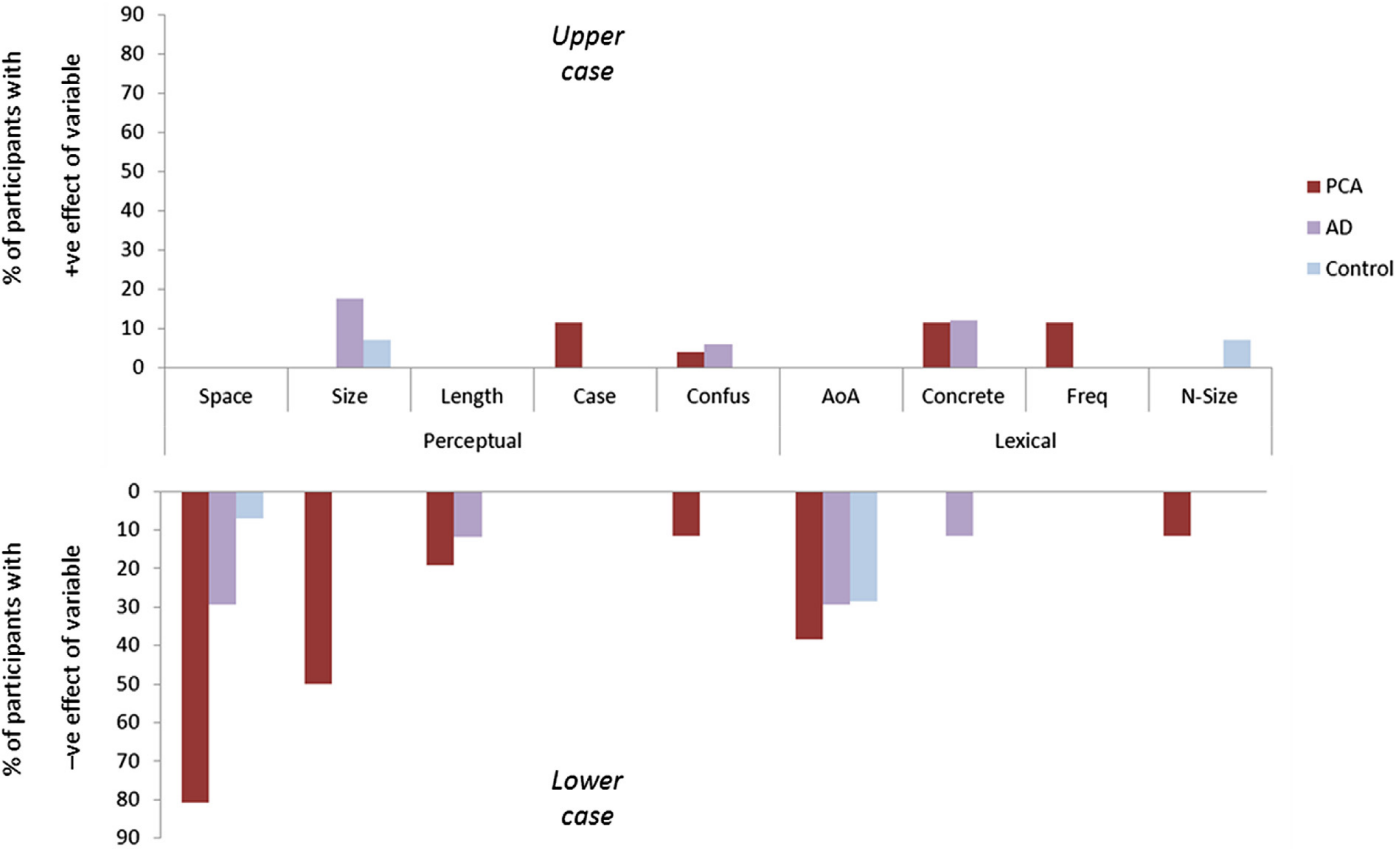
Proportion of participants in each group who show an effect of each variable on either latency or accuracy at the individual level.

The proportion of participants in each group whose reading accuracy or speed was predicted by one or more variables at the individual level is shown in Fig. 2. Increased font size reduced reading accuracy or speed in 46% of the PCA group, but increased reading speed in 18% of the tAD and 7% of the control group.

###### 3.1.1.2.1 Between-group accuracy

As described above, differences in accuracy between the PCA and tAD groups were modelled using mixed-effects logistic regression including as covariates reading variables that were statistically significant at the group level for either PCA or tAD groups. These variables were spacing, size, order, AoA, frequency and length. There were significant interactions between diagnosis and spacing (accuracy: *z* = 2.5, *p* < .05; latency: *z* = −8.6, *p* < .001) and diagnosis and size (accuracy: *z* = 2.8, *p* < .01; latency: *z* = 2.8, *p* < .01), with increased spacing and size leading to lower accuracy in the PCA group; none of these interactions could be accounted for by any of the behavioural correlates.

There was no evidence of a group difference in overall reading accuracy after adjusting for participants' composite scores of the following covariates of interest: visuoperceptual, visuospatial or early visual function, or the A cancellation task; these scores were better predictors of reading accuracy than diagnosis whether included individually or simultaneously in a regression model. The following nuisance variables, including markers of disease severity (MMSE scores, disease duration), nonvisual indicators of executive function (digit span backwards) or single letter recognition performance could not account for group differences in accuracy. This suggests that the between-group differences in overall accuracy were driven particularly by poor early visual, visuoperceptual and visuospatial abilities.

Given the possible role of crowding in limiting reading ability (Crutch and Warrington, 2009, Yong et al., 2013), we conducted a *post hoc* analysis evaluating the extent to which crowding measures accounted for the group difference relative to other measures of early visual processing. A composite [labelled Early visual processing (excluding crowding)] was calculated with the omission of the crowding task score; unlike the composite score for Early visual processing which included measures of crowding, this composite did not account for the between-group difference.

###### 3.1.1.2.2 Between-group latency

Differences in latency were modelled using a mixed-effects linear regression analysis of latency data for the PCA and tAD groups including as covariates reading variables that were significant at the group level for either PCA or tAD groups (spacing, size, order, AoA). There was no evidence of a group difference in overall reading speed after adjusting for participants' composite scores on tests of visuoperceptual function. None of the nuisance variables (disease duration, composite scores, MMSE, digit span backwards, A cancellation, single letter processing tasks) could account for group differences in overall reading latency.

##### 3.1.1.3 Individual differences in accuracy and latency

There was a great degree of variability in reading accuracy within the PCA group (range: 19.8–99.5%). 23/26 (88.5%) of the PCA patients performed below the 5th %ile of the control group's accuracy and latency data when reading small unspaced words. Of the three patients whose reading ability was within the normal range of the control group, two of these patients are reported in Yong et al. (2013).

##### 3.1.1.4 Error analysis

An analysis of PCA error types revealed 68.9% visual errors, 19.3% miscellaneous errors, 9.6% phonological errors and 2.1% derivational errors. In 23/26 participants the most common errors were visual errors: the other three participants only made one error each, with one making a phonological error and the other two making derivational errors. Within the 23 participants making visual errors, the highest proportions of any other single error type were observed in the following patients: Participant 8: 57 miscellaneous versus 71 visual errors; Participant 5: 15 phonological versus 30 visual errors; Participant 4: 3 derivational versus 18 visual errors.

Of the visual errors, 52.2% of letters read incorrectly were substitution errors, 23.6% were deletion errors and 24.2% were addition errors. 17.2% of visual errors were neglect errors (Ellis et al., 1987). Participant 15 made the most errors in the left (*n* = 7) relative to the right (*n* = 1) side of words, while Participant 24 made the most errors in the right (*n* = 12) relative to the left (*n* = 3) side of words.

#### 3.1.2 Cursive font reading

The PCA group (*N* = 22) made, on average, more errors reading words in cursive than non-cursive font (cursive: Mean = 68.6%, SD = 32.4; non-cursive: Mean = 89.3%, SD = 15.8: *z* = −3.71, *p* < .0005). The tAD group scored too near ceiling to reveal any such differences (cursive: Mean = 96.1%, SD = 7.3; baseline: Mean = 97.1%, SD = 5.0: *p* > .8). The PCA group was significantly worse than the tAD group reading cursive font (*z* = 3.29, *p* < .005).

### 3.2 Background neuropsychology

Mean scores for the PCA and tAD groups and an estimate of their performance relative to normative data sets appropriate for the mean age of each group are shown in Table 2. On tasks without a core visual component, the performance of the PCA group was mostly equivalent to (Concrete Synonyms, Naming, Digit Span forwards) or better than (Short Recognition Memory Test: words) that of the tAD group. PCA patients had lower scores than tAD patients on tests sensitive to parietal dysfunction (Calculation, Digit Span backwards, Cognitive estimates) and on the ‘A’ cancellation task, which is a measure of psychomotor speed involving a prominent visuospatial component.

#### 3.2.1 Visual assessment

PCA patients showed greater impairment than the tAD group on all tests of early visual function (except colour discrimination and single letter naming), visuoperceptual function [except unusual (non-canonical) object perception] and visuospatial processing.

#### 3.2.2 Relationship between neuropsychological performance and perceptual variables

Analysis of PCA reading accuracy and neuropsychological data identified interactions between perceptual variables and measures of visual processing. Patients with poor visuospatial function were particularly inaccurate reading words with increased inter-letter spacing (*z* = 3.64, *p* < .001). Patients with poor early visual and visuoperceptual function were particularly inaccurate reading longer words (early: *z* = 3.53, *p* < .001; visuoperceptual: *z* = 3.08, *p* < .005). MMSE scores or disease duration could not account for any of the interactions between visual processing and spacing or word length.

See Supplementary Table 1 for how individual tests predict overall accuracy and latency in PCA and tAD groups.

### 3.3 Neuroimaging findings

**Fig. 3:**
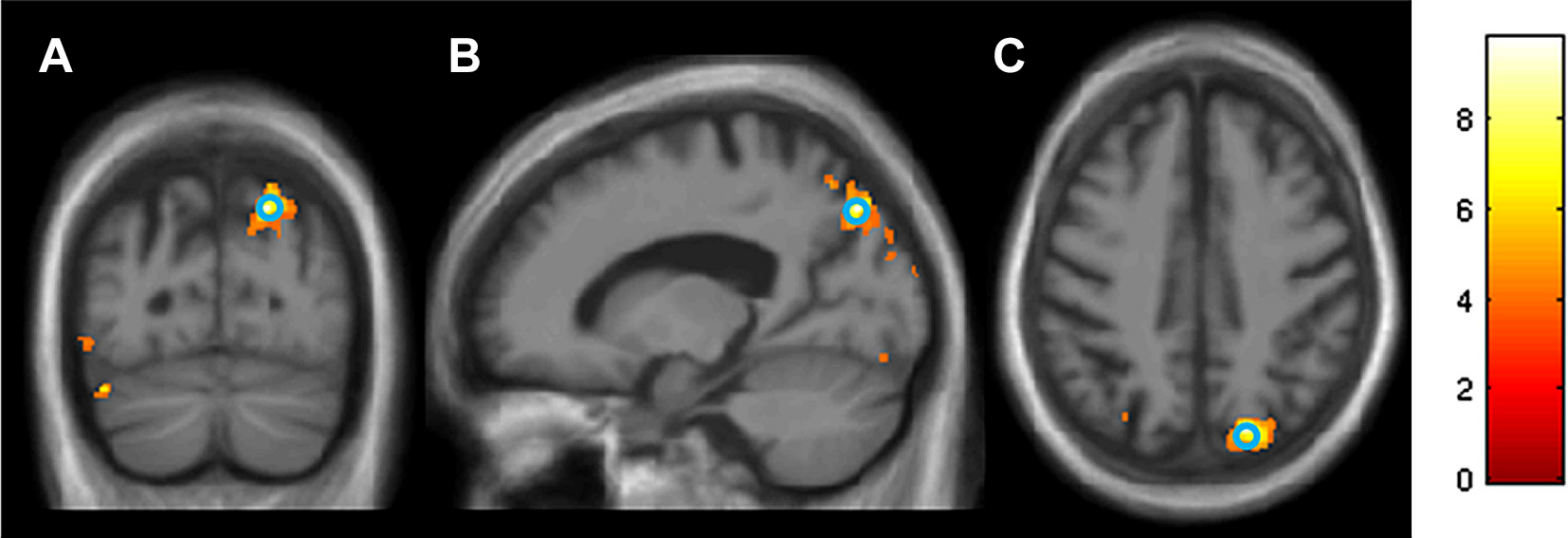
Statistical parametric maps of grey matter volume associated with the difference in accuracy between large and small words in the PCA group. The statistical parametric maps are displayed on coronal (A), sagittal (B) and axial (C) sections of the mean normalized bias-corrected images in MNI space: the right hemisphere is shown on the right on coronal and axial sections. Whole-brain analysis found that, within the PCA group, a greater discrepancy in accuracy between large and small words was associated with reduced grey matter volume in the right superior parietal lobule: *t*-values are displayed below (*p* < .001 uncorrected) with the FWE corrected (*p* = .012) peak circled in blue (peak location: *x* = 18, *y* = −75, *z* = 44). The colour bar shows the *t*-value.

Neuroanatomical associations of reading performance in the PCA group are shown in Fig. 3. In order to identify grey matter associations with reading ability, accuracy discrepancy scores between levels of reading variables which significantly predicted overall reading accuracy in PCA (Large *vs* Small, Spaced *vs* Unspaced, High *vs* Low AoA, High *vs* Low Frequency) were used as behavioural indices. In the PCA group, a greater inverse size effect (lower accuracy for reading large rather than small font size words) was associated with lower grey matter volume in the right superior parietal lobule after correcting for multiple comparisons over whole-brain volume (*p* = .012). There was no evidence of statistically significant associations between grey matter volume and the other three variables tested (spacing, AoA, frequency) in this group.

## 4 Discussion

The current study aimed to better characterise single word reading in PCA and understand the relationship between reading and other visual processes by examining reading of words in which inter-letter spacing, font size, length, font type, case and confusability were varied systematically. On average, the PCA group was considerably less accurate and slower than the tAD or healthy control group, with the tAD group demonstrating slower but not significantly less accurate performance than controls. PCA reading accuracy was predicted by the perceptual variables of letter spacing, size and length plus the lexical variables of AoA and frequency. Similarly, PCA reading speed was predicted by letter spacing, size and AoA. The perceptual complexities of cursive font also had an adverse effect on PCA reading performance whilst overall case and confusability effects were not detected. In contrast, no perceptual variables were predictive of reading accuracy in the tAD or control groups (with high or ceiling level performance in most individuals). Letter spacing, AoA and word order were the only variables which predicted reading speed in both tAD and control groups.

A further prominent difference between the PCA and tAD groups was the *direction* of the size effect. Increasing font size significantly *reduced* accuracy and/or slowed reading for half the PCA participants (46%), whilst larger text improved reading speed overall in the healthy control group and for the minority of tAD participants who showed a size effect (18%). Voxel-based morphometry (VBM) whole-brain analysis within the PCA group found that this size effect (less accurate reading of large than small font size print) was associated with lower grey matter volume in the right superior parietal lobule. VBM analysis within the PCA group did not find significant associations between effects of the other perceptual or reading variables that predicted reading performance and grey matter volume.

The impact of perceptual variables on reading performance and preponderance of visual errors (69%) are unsurprising given that visual impairment is the defining feature of the PCA syndrome. Of greater neuropsychological interest is the determination of which aspects of visual processing are associated with this pattern of reading dysfunction, and the interaction between these processes and text manipulations employed in the current study. We attempted to evaluate which behavioural covariates (including those derived from the detailed visual assessment) might contribute towards reading dysfunction by accounting for the discrepancy in performance between the PCA and tAD groups. PCA patients' inferior reading accuracy relative to tAD patients could not be accounted for by generic markers of disease severity (MMSE, disease duration) but was significantly associated with performance on all three visual covariates (early visual, visuoperceptual and visuospatial processing). However, the early visual processing covariate only predicted accuracy when this composite score included a measure of visual crowding. Furthermore, PCA patients exhibiting greater crowding effects were less accurate reading longer words; assuming increased numbers of letters in longer words operate as multiple flankers, this is consistent with observations of elevated flanker numbers leading to more prominent crowding effects (Poder & Wagemans, 2007). Regarding reading latency, the discrepancy in performance between PCA and tAD patients could only be accounted for by poor visuoperceptual ability. The specific effects of letter spacing and size also could not be accounted for by any of the behavioural covariates, suggesting it is the combination of visual deficits at multiple levels of the visual system which give rise to the observed and distinctive pattern of reading seen in PCA.

Before considering the overall classification of reading impairment in PCA, we discuss possible explanations for the considerable impact firstly of letter spacing and secondly of font size upon patients' reading of the current set of perceptually manipulated words. First, letter spacing was included as one of the perceptual text manipulations in the current investigation because previous case studies had shown its influence upon both single letter and word identification (Crutch & Warrington, 2009). This study revealed optimal letter spacing is partially task dependent. With flanked letter identification, performance was significantly improved by inserting 2 spaces between letters (mean centre-to-centre spacing = 1.52°) as compared with normal presentation text (0 spaces; mean centre-to-centre spacing = .86°). With word reading a U-shaped function was obtained; performance improved when inter-letter spacing was increased from .78° to 1.21°, an effect attributed to a reduction in crowding, but declined again when spacing increased to 2.27°, because increasing spacing past a given point damages whole-word form and parallel letter processing. In the current study, values of .86° (unspaced) and 1.52° (spaced) were selected to maximise individual letter identification ability. However the results, which show significantly *worse* PCA reading performance in the spaced condition, suggest that any benefits in reduced crowding of individual letter identities was outweighed by inevitable increases in the visual angle subtended by the outmost letters within perceptually longer words. Nonetheless, PCA patients showed significantly greater spacing effects than the tAD or control groups, raising questions about the mechanism underpinning the ability to read spatially distributed words.

It has been proposed that failure to achieve parallel letter processing due to presentation of text in unfamiliar formats invokes involvement of dorsally-mediated reading strategies such as serial letter scanning (Braet and Humphreys, 2007, Hall et al., 2001). Reading words with increased inter-letter spacing has been associated with the engagement of parietal lobes in healthy individuals (Cohen, Dehaene, Vinckier, Jobert, & Montavont, 2008), and double spacing has been found to disrupt reading in a patient with occipitoparietal lesions (Vinckier et al., 2006). The current investigation found that PCA patients with poor visuospatial processing were particularly inaccurate when reading spaced words. If reading spaced words demands support from dorsally-mediated reading strategies and/or involves greater visuospatial demands, the vulnerability of dorsal systems in PCA (e.g., Lehmann et al., 2011, McMonagle et al., 2006) might account for these reading deficits. The failure of dorsal-parietal systems in reading unfamiliar text may also account for the PCA group's disproportionately poor reading performance for cursive font, especially as difficult-to-read handwriting has been shown to activate parietal networks in healthy individuals (Qiao et al., 2010). Another possibility is that impaired reading of words with increased inter-letter spacing (or in cursive font) might result from a ventral deficit, possibly a disrupted word-form system, which could accommodate word processing under familiar but not unfamiliar presentation.

Turning secondly to the impact of font size, the PCA group's better reading performance with small rather than large words was not only counter-intuitive but also in direct contrast to size effects seen overall in the control group and in a small number of tAD patients. This size effect may be attributable to what has been termed a (spatial) restriction in the effective visual field, which occurs in right-brain-damaged individuals when the processing demands of more centrally presented stimuli/tasks exhaust available attentional capacity (Russell et al., 2004, Russell et al., 2013). In the current task, though matched for overall form, large font words extend further into the periphery than small print words (this is also the case for spaced as compared with unspaced words as varied in the inter-letter spacing condition). As noted above, grey matter volume analysis in the PCA group found an association between the discrepancy in accuracy between large and small words and grey matter volume in the right superior parietal lobule. This localisation is in keeping with previous studies of peripheral spatial attention. Parieto-occipital damage has been associated with reduced perception and localization within the visual periphery (Michel and Henaff, 2004, Pisella et al., 2009, Rossetti et al., 2005), and greater activation in the superior parietal lobule has been found for stimuli in peripheral vision which were actively attended during an orientation discrimination task (Vandenberghe et al., 1996) or when participants shifted attention towards peripheral vision relative to maintaining attention at fixation (Corbetta et al., 1993).

A potentially complementary explanation of the size effect in PCA is that reading larger words increases the demand for multiple saccades and spatial shifts in attention. fMRI studies have identified saccade-related activation in the superior parietal lobule (Medendorp et al., 2005, Merriam et al., 2003, Sereno et al., 2001), while the superior parietal cortex has been associated with shifting rather than sustained attention (Kelley et al., 2008, Molenberghs et al., 2007, Vandenberghe et al., 2001). As previous studies have identified reaching, perceptual and localization deficits in the peripheral vision of superior parietal lobule lesion patients maintaining central fixation (Pisella et al., 2009, Rossetti et al., 2005, Wolpert et al., 1998), it is unlikely that deficits in integrating information across multiple saccades can completely account for the inverse size effect.

Beyond the impact on single word recognition in PCA, the inverse size effect documented in these patients also has implications for reading at and above the sentence level. Any restriction in the effective visual field would limit the perceptual span and parafoveal preview benefit (Hyona et al., 2004, McDonald, 2006, Rayner, 1998) and might inhibit the ability to move between consecutive lines of text, as has been previously observed in PCA (Ross et al., 1996) and in a patient with Balint's syndrome (Michel & Henaff, 2004). An interesting comparison group is patients with retinitis pigmentosa, a condition involving a progressive pigmentary degeneration of the retina, often resulting in restricted central area of vision, or “tunnel vision” (Madreperla, Palmer, Massof, & Finkelstein, 1990). Increased reading speed has been observed in patients with retinitis pigmentosa when reading words of reduced font size (Sandberg et al., 2006) and words presented in negative polarity, i.e., white text on a black background (Ehrlich, 1987). Reverse polarity presentation may be a particularly promising manipulation, given its ameliorating effect on crowding in both PCA patients and healthy individuals (Chakravarthi and Cavanagh, 2007, Crutch and Warrington, 2007, Crutch and Warrington, 2009, Kooi et al., 1994). Presentation methods that reduce the need for visuospatial processing in reading, such as rapid serial visual presentation or horizontally scrolling text (Leff & Behrmann, 2008) may be also beneficial in limiting visual disorientation.

One important caveat to the current group study is that reading is not uniformly impaired in PCA in all conditions. Two PCA patients in the current sample demonstrated preserved reading of normally presented (small, unspaced) words and exhibited normal accuracy and speed on several other word corpora despite exhibiting impairments on almost every measure of visual processing (Yong et al., 2013). The reading ability of these patients indicates that many forms of early visual, visuoperceptual and visuospatial impairment are not necessarily causally linked to reading dysfunction; instead, their performance suggests that deficits in orthographic processing may arise from damage to a specific form of processing or neural substrate (Roberts et al., 2013; Warrington & Shallice, 1980) rather than a result of general visual impairment (Behrmann et al., 1998, Mycroft et al., 2009). Overall analysis of the PCA group revealed an effect of word length on reading accuracy, but not reading speed. There was a length effect on reading speed in two individual PCA patients, but the absolute mean increase in reading latency for each additional letter (Participant 17: 36 msec/letter; Participant 26: 9 msec/letter) was an order of magnitude smaller than that reported in previous accounts of letter-by-letter reading (90–7000 msec/letter: Fiset et al., 2005, Mycroft et al., 2009).

The current findings suggest that not one but a combination of deficits are associated with the acquired peripheral dyslexia observed in PCA. Overall, poor reading accuracy is associated with deficits in early visual processing, particularly including visual crowding, and poor visuoperceptual and visuospatial ability. However, these deficits are not causally related to a universal impairment of reading (as shown by preserved reading for small, unspaced words in some patients) but rather are (con)text specific (being particularly evident for large, spaced or crowded lengthy words). The vulnerability of dorsal systems in PCA may account for disproportionate difficulties reading text which eludes ventrally-mediated parallel letter processing: that is, words written in unfamiliar formats, such as text with double spacing or cursive font. Poor visuospatial ability and restrictions in the effective visual field as a consequence of parietal atrophy may also explain the inverse size effect. The profile of reading impairment in PCA does not align with any classical subtypes of peripheral dyslexia (e.g., pure alexia, neglect dyslexia), underlining why previous investigators have coined the term “apperceptive alexia” to capture the combination of contributory deficits (Mendez et al., 2007). However, further to the suggestions of Mendez et al. (2007): that apperceptive alexia might be attributable to visuoperceptual and visuospatial deficits, the current findings also indicate the role of early visual processing deficits, particularly visual crowding, in contributing towards poor reading. Clinically, the findings also provide directions as to the design of presentation conditions that may maximise and sustain reading ability through the early years of the disease.
