# Supplementary material for: (Con)text-specific effects of visual dysfunction on reading in posterior cortical atrophy
Source: Cortex. 2014 Aug;57:92–106. doi: 10.1016/j.cortex.2014.03.010 (PMC4194349; doi:10.1016/j.cortex.2014.03.010)
Supplement: Supplementary file 1 [file mmc1.docx]

Supplementary Table 1. Reading performance in PCA and tAD predicted by disease severity and performance on measures of early visual, visuospatial and visuoperceptual processing. Measures that significantly predict reading performance are highlighted in green, measures that show a trend towards predicting reading performance are highlighted in light green.

|  |  | **Disease Severity** | | **Early visual processing** | | | **Visuospatial processing** | | **Visuoperceptual processing** | | | |
| --- | --- | --- | --- | --- | --- | --- | --- | --- | --- | --- | --- | --- |
|  |  | Disease Duration | MMSE | Figure-ground | Shape Discrimination | Crowding | Number location | Dot counting | Object decision | Fragmented letters | Usual views | Unusual views |
| PCA | accuracy | *z=-2.43, p<.05* | *z=3.54, p<.001* | *z=3.32, p<.005* | *p>.1* | *z=4.47, p<.001* | *z=4.53, p<.001* | *z=2.66, p<.01* | *z=4.95, p<.001* | *z=3.52, p<.001* | *z=2.56, p<.05* | *z=3.31, p<.005* |
|  | latency | *z=2.03, p<.05* | *z=-2.54, p<.05* | *p>.5* | *p>.6* | *z=-2.08, p<.05* | *z=-2.29, p<.05* | *p>.3* | *p>.1* | *z=-1.71, p=.087* | *p>.7* | *z=-1.97, p<.05* |
| tAD | accuracy | *p>.3* | *p>.5* | *p>.9* | *p>.7* | *z=-4.63, p<.001* | *p>.7* | *p>.6* | *z=2.20, p<.05* | *z=2.38, p<.05* | *z=1.89, p=.059* | *p>.3* |
|  | latency | *p>.7* | *p>.5* | *p>.6* | *p>.8* | *collinear* | *p>.7* | *z=-1.75, p=.081* | *z=-2.24, p<.05* | *z=-2.31, p<.05* | *z=-2.38, p<.05* | *p>.6* |
